# Reactome and the Gene Ontology: digital convergence of data resources

**DOI:** 10.1093/bioinformatics/btab325

**Published:** 2021-05-08

**Authors:** Benjamin M Good, Kimberly Van Auken, David P Hill, Huaiyu Mi, Seth Carbon, James P Balhoff, Laurent-Philippe Albou, Paul D Thomas, Christopher J Mungall, Judith A Blake, Peter D’Eustachio

**Affiliations:** Division of Environmental Genomics and Systems Biology, Lawrence Berkeley National Laboratory, Berkeley, CA 94720, USA; Division of Biology and Biological Engineering, California Institute of Technology, Pasadena, CA 91125, USA; The Jackson Laboratory, Bar Harbor, ME 04609, USA; Division of Bioinformatics, Department of Preventive Medicine, University of Southern California, Los Angeles, CA 90033, USA; Division of Environmental Genomics and Systems Biology, Lawrence Berkeley National Laboratory, Berkeley, CA 94720, USA; Renaissance Computing Institute, University of North Carolina at Chapel Hill, Chapel Hill, NC 27517, USA; Division of Bioinformatics, Department of Preventive Medicine, University of Southern California, Los Angeles, CA 90033, USA; Division of Bioinformatics, Department of Preventive Medicine, University of Southern California, Los Angeles, CA 90033, USA; Division of Environmental Genomics and Systems Biology, Lawrence Berkeley National Laboratory, Berkeley, CA 94720, USA; The Jackson Laboratory, Bar Harbor, ME 04609, USA; Department of Biochemistry and Molecular Pharmacology, NYU Grossman School of Medicine, New York, NY 10016, USA

## Abstract

**Motivation:**

Gene Ontology Causal Activity Models (GO-CAMs) assemble individual associations of gene products with cellular components, molecular functions and biological processes into causally linked activity flow models. Pathway databases such as the Reactome Knowledgebase create detailed molecular process descriptions of reactions and assemble them, based on sharing of entities between individual reactions into pathway descriptions.

**Results:**

To convert the rich content of Reactome into GO-CAMs, we have developed a software tool, Pathways2GO, to convert the entire set of normal human Reactome pathways into GO-CAMs. This conversion yields standard GO annotations from Reactome content and supports enhanced quality control for both Reactome and GO, yielding a nearly seamless conversion between these two resources for the bioinformatics community.

**Supplementary information:**

Supplementary data are available at *Bioinformatics* online.

## 1 Introduction

**Fig. 1.**
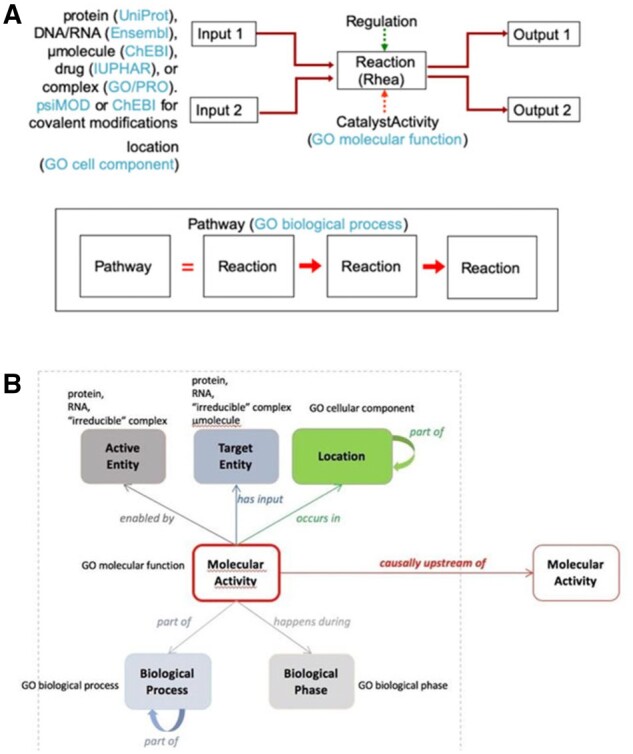
A Reactome reaction (**A**) is the transformation of input physical entities into output ones through changes in their covalent chemistries, their subcellular locations, or their associations with other entities in complexes. Physical entities may catalyze or regulate a reaction. The blue parenthetical notes identify the external resources and ontologies from which information are drawn to annotate reactions. Sequential reactions are connected to form a pathway where the output of an upstream reaction is an input, catalyst or positive regulator of a downstream reaction. A GO-CAM model (**B**) centers on a molecular activity enabled by a single gene product or, inspecific cases, a complex (active entity), acting on a target entity, occurring in a subcellularlocation, comprising a part of a biological process, during a biological phase. Arrows are relations from the RO, and all boxes refer to a class from an ontology(or other stable object identifier). Curved ‘part of’ arrows show nesting of smaller processes or cell parts as modules within larger ones

A biological pathway is an ordered set of molecular steps by which a process such as the metabolism of a small molecule or a signaling cascade is accomplished. Pathway information derived from vast collections of experimental results are available in the widely used Reactome Knowledgebase (Jassal *et al.*, 2020) and in many other data resources (Bader *et al.*, 2006; Rodchenkov *et al.*, 2020). These resources organize molecular details of participating small molecules, proteins and other macromolecules, and complexes and their interactions as inputs, outputs, catalysts and regulators of reactions (Fig. 1A), comparable in detail and organization to the process description (PD) SBGN visualization of a pathway (Le Novère *et al.*, 2009).

A GO annotation is an association between a single gene product and a single GO term (GO Consortium 2000, 2021). The recently developed Gene Ontology Causal Activity Modeling (GO-CAM) framework defines a schema for linking together multiple annotations (as well as additional contextual information) into larger models of biological processes, including pathways (Thomas *et al.*, 2019; see also http://geneontology.org/docs/gocam-overview/, http://geneontology.org/docs/sparql and https://github.com/geneontology/neo#readme). All elements of a GO-CAM model are represented as instances of classes from standard ontologies. The central element in GO-CAM is the molecular activity, an instance of a GO molecular function term; multiple activities are connected by causal relations into a data model (Fig. 1B) that is comparable in detail and organization to the activity flow (AF) SBGN visualization of a pathway (Le Novère *et al.*, 2009).

We will use PD (process description) and AF as a convenient shorthand to refer both to the data structures embodied in Reactome pathways and GO-CAM models, respectively, and to the SBGN visual representations of those data structures. The use of PD and AF representations is discussed more fully in Supplementary File S1.

Here, as a step toward a comprehensive alignment of two leading resources for pathway data, we describe work to convert the PD representation used by Reactome into the AF representation used in GO-CAMs (Thomas *et al.*, 2019). This conversion benefits consumers of structured pathway data. It enhances interoperability, integrationand data exchange between GO, Reactome and other pathway databases, and it supports the ability to leverage the knowledge formally encoded in OBO OWL ontologies to automatically identify inconsistencies and infer new knowledge. Finally, because the conversion process takes as input Reactome’s content in the BioPAX community standard format (Demir *et al.*, 2010, 2013), the process would be generalizable to other pathway data in this format, e.g. Pathway Commons (Rodchenkov *et al.*, 2020).

Previously developed strategies to convert PD to AF representations (Vogt *et al.*, 2013) start with the conversion of each molecular entity to an activity. However, most reactions in Reactome are either controlled by a protein catalyst or represent regulatory binding interactions involving proteins. To preserve this additional, biologically important detail, we describe here a process, Pathways2GO, in which we consider each distinct reaction as a potential activity and apply a set of rules to convert Reactome reactions (BioPAX PD model) to gene product activities and to infer causal relations between them (GO-CAM AF model).

## 2 Materials and methods

### 2.1 Owl representations of GO-CAMs

The GO-CAM structure is achieved as an OWL ‘instance graph’ implemented in the OWL 2 ontology language (Balhoff *et al.*, 2018; Thomas *et al.*, 2019). Nodes in the graph correspond to OWL: individuals and edges to OWL: ObjectProperties drawn from the Relation Ontology (RO—Smith *et al.*, 2005). Evidence for asserted relationships in the graph is captured as OWL 2 annotations using the ECO ontology (Giglio *et al.*, 2019) and appropriate references. Each individual in the graph is assigned a semantic type using an OWL Class. These classes are drawn from a subset of the OBO ontology collection, including GO (GO Consortium 2000, 2021), ChEBI (Hastings *et al.*, 2016), and anatomy ontologies such as CL (https://www.ebi.ac.uk/ols/ontologies/cl; Meehan *et al.*, 2011), DDANAT (http://dictybase.org/Dicty_Info/dicty_anatomy_ontology.html; Gaudet *et al.*, 2008) and EMAPA (http://www.obofoundry.org/ontology/emapa.html; Hayamizu *et al.*, 2015). The classes used to define gene products are drawn from NEO, the Noctua Entity Ontology (https://github.com/geneontology/neo#readme), which integrates identifiers for all of the gene products used in GO-CAMs into a single large OWL ontology, rooted under the ChEBI class ‘information biomacromolecule’ (CHEBI: 33695).

**Fig. 2.**
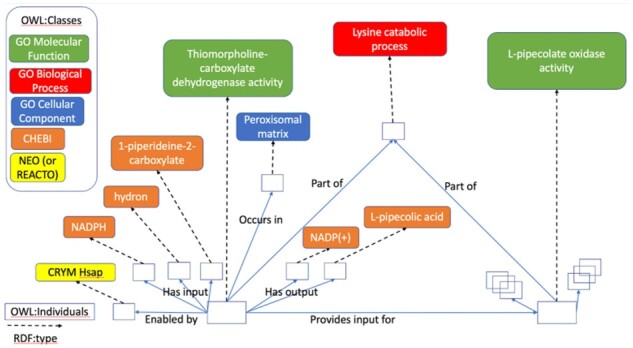
OWL instance graph representing a part of the GO-CAM for the lysine catabolic pathway. Nodes (open rectangular boxes) correspond to OWL: individuals. The ontology term associated with each node and edge in a GO-CAM graph provides its human readable label and its logical definition. The ontologies provide logical definitions for classes and properties that enable use of OWL reasoning to infer new classifications for individuals in GO-CAMs and to check GO-CAMs for logical consistency

As a simple example, Figure 2 depicts the OWL instance graph representing the Reactome reaction ‘CRYM reduces P2C to PPCA’ (R-HSA-5693347), which is part of in the lysine catabolism pathway (R-HSA-71064). This reaction is catalyzed by the CRYM protein, has three inputs: H+ (‘hydron’), P2C and NADPH and two outputs: NADP+ and PPCA. Reactome curators have manually classified the reaction as an instance of thiomorpholine-carboxylate dehydrogenase activity (GO: 0047127) occurring in the peroxisomal matrix (GO: 0005782) and part of in the lysine catabolic process (GO: 0006554).

### 2.2 The Reactome entity ontology, REACTO

As a result of these differences in the scope of entity representation within Reactome and the GO, we made a pragmatic choice to take Reactome entities that do not map to existing GO-CAM ontologies, and represent them in a new ontology, REACTO, that defines a class for each of the unmapped entities in Reactome. This ontology, like the NEO ontology used in the GO-CAM editorial interface, contains class references for physical entities including logical definitions and links to canonical identifier systems such as UniProt and ChHEBI.

All of the classes needed to define the instances in the OWL instance graph must be present in the imported ontology collection in order for them to be represented in the GO-CAM graph (Fig. 2). The current default ontology collection employed by the GO Consortium for building GO-CAMs is extensive, including more than a million classes and more than two million logical axioms, but does not contain classes for many of the physical entities referred to in Reactome. Specifically:

1. Reactome makes extensive use of protein complexes in their pathway representations, describing their formation, dissociation and activities. In contrast, the GO terms generally apply to the activities of individual gene products.
2. Reactome creates a new entity for each combination of physical entity and location. For example, the Reactome id for cytosolic ADP is R-ALL-29370 while nuclear ADP is R-ALL-113582.
3. Reactome pathways often use modified forms of proteins while GO annotations usually refer to a gene or the canonical form of a gene product. For example, the Reactome pathway ‘MAP2K and MAPK activation’ makes use of the entity ‘S-Farn-Me-PalmS KRAS4A’ (R-HSA-9647915) which refers specifically to the GTPase KRAS protein (UniProt: P01116) with its carboxyterminal three residues removed and the cysteine residues at positions 179 and 186 covalently modified by S-palmitoylation and S-farnesylation, respectively.
4. Reactome uses a set-based representation in many of their reactions as a shorthand for groups of similarly acting physical entities, e.g. glucokinase and hexokinases (https://reactome.org/content/detail/R-HSA-450097). We represent Reactome sets as Unions (logical OR) in OWL Class expressions in the REACTO ontology (Supplementary Figs S2 and S3). This representation signifies that any member of the set can act in the modeled reaction.

### 2.3 Conversion process

Given a pathway expressed in the BioPAX (level 3) exchange format, the procedure for generating a GO-CAM proceeds in eight steps, described in detail in Supplementary File S2—Pathways to GO conversion procedure and documented online: https://github.com/geneontology/pathways2GO.

1. Generate or identify an OWL ontology that contains a class representation of each of the physical entities in the BioPAX file.
2. Construct the OWL: individuals for the model with default types, such as GO: biological process or GO: molecular function.
3. Use semantic annotations provided as BioPAX: RelationshipXrefs to add deeper classifications such as more specific GO biological process terms for BioPAX: pathway nodes and GO molecular function terms for BioPAX: BiochemicalReactions.
4. Attempt to infer classes for individuals with no provided semantic annotations.
5. For each GO-CAM activity node, link to the physical entities relevant to that activity.
6. Infer location information assertions for all activity nodes.
7. Infer causal relations between activity nodes.
8. Convert noncatalytic entity regulators to binding activity nodes.

## 3 Results

### 3.1 Deriving GO-CAM models from Reactome pathways

Reactions in BioPAX are represented as conversion events, i.e. transformations of pools of molecules by modification (e.g. chemical reactions of small molecules, covalent modifications of proteins, formation of complexes), or transport into different subcellular locations. A BioPAX pathway is a directed network of these reactions.

The central premises of the Pathways2GO conversion process are that conversion events within pathways correspond to molecular activities (annotated as GO molecular functions) and that the attributes of a Reactome reaction can be mapped to those of a complete GO-CAM activity unit. A complete activity unit, like a Reactome reaction, has attributes that specify:

1. The nature of the activity (a GO molecular function).
2. The entity that enables the activity (a gene product or complex of them).
3. The entities that serve as inputs and outputs of the activity (small molecules, proteins, complexes).
4. The location in the cell where the activity occurs (a GO cellular component).
5. If the activity is a form of transport, the location of the transported entity at the beginning and end of the activity.
6. The biological process (pathway) of which the activity is a part (GO biological process).
7. At least one causal connection to another activity unit.

To create a GO-CAM activity unit, physical entities are tagged with their reference identifiers [e.g. UniProt (UniProt Consortium, 2017), ChEBI (Hastings *et al.*, 2016) and GO molecular functions and cellular components]. These constructs are joined with semantic relations from the RO (https://www.ebi.ac.uk/ols/ontologies/ro) between a gene product’s molecular function and its location: a gene product’s activity ‘occurs in’ (BFO: 0000066) a location that corresponds to a GO cellular component.

**Fig. 3.**
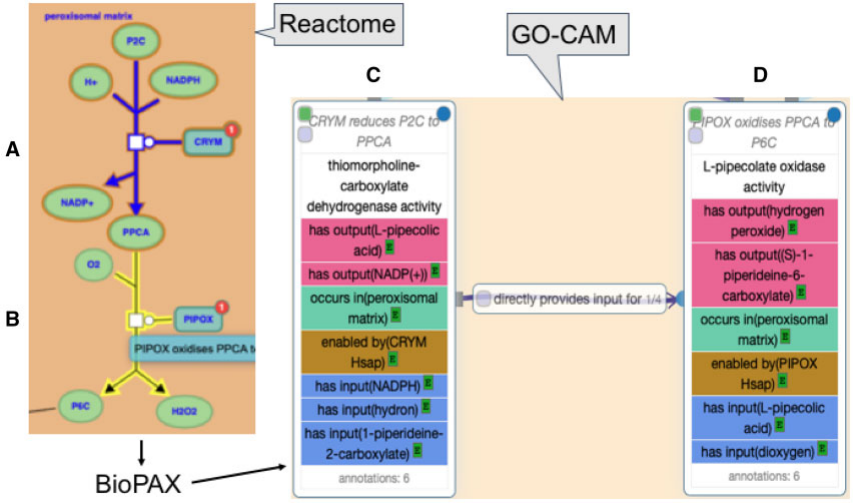
Conversion of the Reactome representation of two reactions, **A** and **B**, in the pathway of human lysine catabolism to GO-CAM activity units, **C** and **D**, respectively, preserving the type of each reaction, subcellular locations of physical entities and the causal relationship between the two reactions/activities. The BioPAX and .ttl files generated in the course of converting Reactome reaction A (R-HSA-71064) to GO-CAM activity unit C are available here (https://reactome.org/PathwayBrowser/#/R-HSA-71064&DTAB=DT) and (https://github.com/geneontology/noctua-models/blob/master/models/R-HSA-71064.ttl), respectively

These GO-CAM activity units (physical entities + molecular function + cellular component) can be further identified as ‘part of’ (BFO: 0000050) a biological process (GO biological process term). They can also be causally linked to other activity units associated with the same process with RO relations such as ‘directly positively regulates’ (RO: 0002629), ‘directly negatively regulates’ (RO: 0002630) and ‘directly provides input for’ (RO: 0002413) (Fig. 3).

Details of the approaches used to extract this information from BioPAX-formatted pathways are in Supplementary File S2—pathways to GO conversion procedure.

### 3.2 Source data

All Reactome-derived GO-CAM models were generated using the BioPAX level 3 export of Version 73 of the Reactome knowledgebase, released on June 17, 2020. This export contained 2423 Human Pathways composed of 13 248 reactions. We filtered out all pathways from the

Disease branch of the Reactome knowledgebase as well as all reactions involving a drug. Following these filters, the input to the Pathways2GO conversion process consisted of 1817 human pathways and 11 570 reactions.

### 3.3 Reaction-level results

**Fig. 4.**
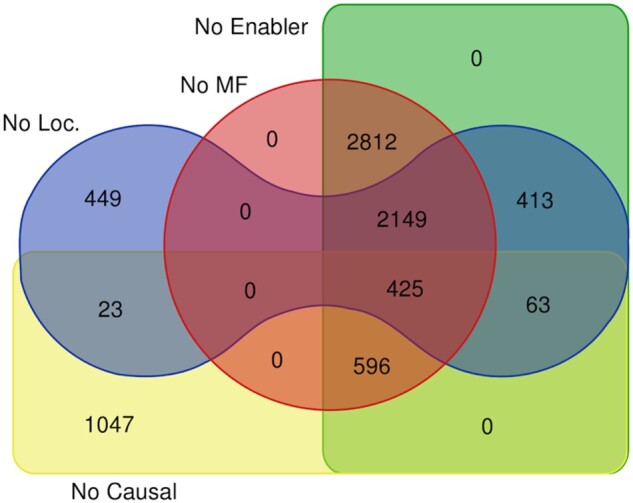
This diagram catalogs classes of missing information in the 7986 incomplete activity units converted from Reactome reactions, specifically: molecular function (MF, red circle), enabling entity (Enabler, green rectangle), location/cell component (Loc, blue toroid), causal connection (Causal, yellow rectangle). Also, 5572 converted reactions could not be assigned a specific GO biological process term but instead were linked to the root term ‘biological process’. All such reactions are parts of Reactome pathways not themselves assigned a specific GO biological process term, a gap in current Reactome curation practice

The goal of the conversion process is to translate the Reactome (PD-like) representation of each reaction into an activity unit suitable to be a node in a GO-CAM (AF-like) representation. All 11 570 of the reactions that were processed were successfully converted into GO-CAM activity units. 3593 of these activity units were complete, demonstrating that the Reactome—BioPAX representation of a reaction can provide all information needed to completely specify a GO-CAM activity unit and the conversion framework can successfully extract it. Causes of activity unit incompleteness and the numbers due to each cause are shown in Figure 4.

The molecular function of a GO-CAM activity unit must be enabled by a single gene product, or by a complex (only if the activity cannot be ascribed to a single gene product, and therefore is an emergent property of the complex). The causal connections between activity units can be derived from their molecular functions, which thereby supports efficient derivation of compact, consistent AF models from GO-CAMs. For example, in Figure 3, the thiomorpholine-carboxylate dehydrogenase activity of CRYM enzyme (Fig. 3C) enables the production of the small molecule pipecolic acid, a required chemical input of the following activity unit (Fig. 3D) in the process of lysine catabolism; therefore, the causal relation between activities 3C and 3D is provides_input_for. The GO-CAM requirements are satisfied by Reactome reactions involving catalysis or transport, and all such Reactome reactions yielded GO-CAM activity units with fully specified molecular function and enabled_by attributes. The Reactome model of a binding or dissociation event, however, treats the participating physical entities as equals even in cases like ligand–receptor interactions where identification of an enabling binding partner could be straightforward. The 11 580 processed reactions included 4158 binding and 416 dissociation events (different numbers of inputs and outputs with no associated catalysis). These binding and dissociation reactions account for a large number of activity units with no associated enabler. They can nevertheless be causally ordered in an AF by noting the overlap of their inputs and outputs with entities associated with upstream and downstream activity units.

A second group of reactions that lack an enabler are shorthand ‘black box’ reactions like R-HSA-1912415 ‘NOTCH3 gene transcription’, created to allow annotation of the regulatory roles of transcription factors and cofactors in *NOTCH3* gene expression without requiring all of the steps of transcription and translation to be represented explicitly. The resulting events can be precisely located in a causal reaction network but lack associated enablers.

To provide a molecular function attribute for these and a small number of additional reactions for which no enabling gene product was asserted or inferred, we have created a new REACTO class, molecular event. The class is added as a superclass of both molecular activities enabled by gene products (molecular functions) and ones enabled in an unspecified way.

Three thousand five hundred and twenty-nine of the incomplete reaction conversions were not assigned a location. The location assigned to a Reactome reaction spans all of the locations of all of its participants. Thus, uptake of an extracellular small molecule would have extracellular, plasma membrane and cytosolic locations. A GO-CAM activity unit may have only a single location. While activity units for transmembrane transport and signaling reactions are thus formally incomplete, the locations of the inputs, outputs and enablers associated with the location-less activity unit are preserved, so no information is lost.

### 3.4 Model-level results

Pathways represent the flow of information from one reaction to the next. A successful conversion to the GO-CAM representation should preserve the connectivity between reactions (converted to activity units). Further, it should identify appropriate causal relationships to define the meaning of the connections, e.g. distinguishing reactions that ‘directly negatively regulate’ downstream reactions from those that ‘provide direct input for’ the next reaction. The Pathways2GO conversion process achieves these goals for all 1817 of the Reactome pathways that describe normal human biology.

The BioPAX representation of Reactome pathways captures reaction–reaction connections using the BioPAX: nextStep relation. As described in detail in Supplementary Methods, each of these connections was converted to one of four RO relations: ‘causally upstream of’ (RO: 0002411) (5435 instances), ‘directly provides input for’ (RO: 0002413) (5273 instances), ‘directly positively regulates’ (RO: 0002629) (878 instances) or ‘directly negatively regulates’ (RO: 0002630) (140 instances). Of the 11 753 reaction–reaction relations in the 1817 converted pathways, all but 27 were converted to causal relations in the GO-CAM framework. The missing relations linked reactions to reactions from disease pathways or drug reactions that had been filtered out of the analysis.

### 3.5 Access to the models

The current GO-CAM model of Reactome pathway R-HSA-### can be accessed via URL: http://model.geneontology.org/R-HSA-###, where R-HSA-### represents the core identifier for a Reactome pathway. Suffixed release identifiers R-HSA-###.# will not resolve, nor will identifiers for individual Reactome reactions. A full set of models is generated for pathways from each Reactome release. A pathway that contains only subpathways are represented by a single node in the GO-CAM model. A pathway that contains reactions are represented by activity units for each of them. Previous versions of models are archived within the GitHub repository at https://github.com/geneontology/noctua-models.

## 4 Discussion

In earlier work, we manually aligned a small group of human metabolic pathways from Reactome with GO biological processes (Hill *et al.*, 2016). Here, we describe a computational process, Pathways2GO, that automates this alignment, converting each of the 1817 pathways that comprise Reactome’s description of normal human biology, into a GO-CAM. Each of these models represents the actions of gene products on one another and on small molecules, causally linked to represent the full sequence of molecular events in the Reactome pathway from which the GO-CAM was derived.

This work has driven the extension of the GO-CAM model to support features of cellular and molecular biology that extend the scope of GO. While GO annotation is classically focused on the properties of single gene products, many human biological processes depend on the activities of covalently modified forms of proteins or proteoforms, often with different forms of the same protein having different activities and of multicomponent complexes. Further, it is often useful to group related entities that share an activity into a set. To support the incorporation of these Reactome-derived entities into GO-CAM activity units, we built a new ontology, REACTO, to contain these classes of physical entities and the relationships among them.

The REACTO ontology includes features that correspond to those of the Protein Ontology (PRO) (Natale *et al.*, 2017). PRO is a well-recognized community resource, and addition of PRO instance identifiers to Reactome proteoforms, complexes and sets and adaptation of Pathways2GO will simplify the generation of GO-CAMs from Reactome pathways and the extension of the conversion process to other pathway resources and to model organism pathways. More important, it provides a robust, ontologically rigorous mechanism to annotate functions of physical entities that are not simply canonical gene products.

Even with the addition of REACTO, however, Reactome-derived GO-CAM activity units sometimes lack attributes needed for completeness (Fig. 4). Pilot studies suggest that, in many cases, the Reactome: GO alignment provided by the GO-CAM models will allow GO reasoning tools to be applied to recover available information missing from Reactome annotations. For example, examination of a representative sample of Reactome events that lack a GO biological process attribute generated by other resources could provide plausible biological process attributes, allowing fast semiautomated recuration of the Reactome events to supply the missing information. This process will improve the consistency and completeness of both resources, and serve as a model for incorporation of data from other pathway resources into a community GO-based annotation resource.

The missing attributes also highlight broader aspects of the Reactome data model, the GO ontology structure, or both, that could be modified to allow better alignment and annotation in both resources. A review of these illustrates the uses and limitations of the current set of Reactome-derived GO-CAM models, and suggests ways in which Reactome and GO can be better aligned to provide a unified bioinformatics resource and to serve as a template for integration of material from still other resources.

For example, the Reactome annotations of gene expression and protein degradation events by their nature properly lack input and output entities, respectively. Work is in progress to develop a reaction typology to identify such ‘shorthand’ reactions and distinguish kinds of them from enzyme-catalyzed reactions, transport reactions, binding reactions and so forth. Both in Reactome and in Pathways2GO, the typology will support creation of varied forms of activity units with checks for annotation consistency and completeness.

Another example of where Reactome/GO alignment could be improved is the annotation of proteins that span a membrane with domains accessible from the spaces on both sides of the membrane, given the current GO-CAM requirement that a gene product enables an activity in a single location. A possible resolution is to adopt the ‘surrounded by’ attribute for GO cell_component terms (e.g. nucleoplasm surrounded_by nuclear envelope surrounded_by cytosol surrounded_by plasma membrane surrounded_by extracellular space). already implemented in Reactome (Jassal *et al.*, 2020). In both GO and Reactome, molecular interactions require direct physical contact among participants, so this relationship will enable a test to confirm that participants are in the same or adjacent locations. Applied to the annotation of compartment-spanning entities, we can define a principal location for such an entity, and apply the adjacency test to validate the locations of interacting entities. The result would be an annotation, consistently in Reactome and GO-CAM that followed a correct path through cell compartments, e.g. the binding of an extracellular ligand to a receptor associated with the plasma membrane enables the receptor, still in the membrane, to bind a cytosolic effector protein.

A third example is identification of causal connections between pathways. Many such connections have been manually annotated in Reactome as preceding/following event relationships between reactions. For example, the generation of cytosolic pyruvate as an output of glycolysis is easily linked to the array of pathways that require it as an input or regulator. Where these causal relationships link activity units within a GO-CAM, they are accurately captured. The GO-CAM formalism at present cannot represent such relationships between GO-CAMs. Pilot studies suggest, as expected, that if such links could be generated, they would allow the generation of integrated molecular views of complex cellular processes (B. Good and D. Hill, unpublished data).

A fourth, more complex, example arises from the GO-CAM requirements that (1) the molecular function of an activity unit must be enabled by a single gene product (or in specific cases by a complex) and (2) even binding and dissociation reactions must be represented as having a primary enabler that executes that activity. To fully align Reactome and GO-CAM representations of processes, it will be necessary to identify ‘enabling’ participants in events involving association and dissociation of gene products. Manual examination of activity units derived from Reactome binding reactions, the largest affected group of activity units, suggests that enabling relationships generally exist. For example, the binding reaction between WNT and its receptor could be represented by WNT as the enabler of a ligand activity acting on the FZD receptor. Such relationships could be captured as additional annotations in Reactome, or automatically extracted from manually curated reaction names.

In summary, we have described the mapping of a PD pathway representation, Reactome, onto an AF one, GO-CAM. We have identified a large number of cases in which such a mapping is straightforward and proceeds with no loss of information. Moreover, even where mapping remains incomplete, much information are preserved and the resulting detailed cross-referencing supports easy navigation between the two types of representations, as envisioned in the original description of the SBGN project:

By ignoring processes and entity states, the number of nodes in an AF diagram is greatly reduced compared to an equivalent process diagram. … The drawback is that AF diagrams may contain a high level of ambiguity. For instance, the biochemical basis of a positive or negative influence in a given system is left undefined. For this reason, this type of SBGN diagram should not exist alone; it should be associated, when possible, with detailed entity relationship and process diagrams, and used only for viewing purposes. We expect it will often be possible to generate AF diagrams mechanically from process diagrams and entity relationships (Le Novère *et al.*, 2009).

The use of ontologies in GO-CAM resolves the ambiguities in an AF diagram, and the conversion between Reactome pathways and GO-CAMs results in both AF and PD representations of the same pathways, supporting a broader set of distinct use cases for Reactome pathway information.

## Funding

This work was supported by grants from the National Institutes of Health [U41 HG02273 to support the GO Consortium; U41 HG 003751 to support the Reactome Knowledgebase], and by funds from the Director, Office of Science, Office of Basic Energy Sciences, of the U.S. Department of Energy [Contract No. DE-AC02-05CH11231 to C.J.M. and S.C.].

##  

*Conflict of Interest*: none declared.

## Supplementary Material

btab325_supplementary_data
