# Supplementary material for: Reactome and the Gene Ontology: digital convergence of data resources
Source: Bioinformatics. 2021 May 8;37(19):3343–8. doi: 10.1093/bioinformatics/btab325 (PMC8504636; doi:10.1093/bioinformatics/btab325)
Supplement: btab325_supplementary_data [file btab325_supplementary_data.pdf]

SUPPLEMENT 1 - AF and PD models of biological processes

The focus on reactions in the Reactome data model (Vastrik et al. 2007) versus activities of gene products (proteins and functional RNAs) in GO-CAM (Thomas et al. 2019) leads to an important difference in the treatment of gene products. In Reactome, gene products are treated like any other molecular entity (e.g. a small molecule metabolite). Both covalent and non-covalent (binding) reactions acting on a protein will *transform* it into a new molecular entity (a modified protein, or a complex of multiple proteins). In GO-CAM, gene products, but not other molecules, enable activities (GO molecular functions). Molecular modifications of gene products are represented in terms of their effects on these activities, rather than in terms of transformations into new molecular entities. This difference presents a major challenge in aligning Reactome and GO-CAM. The first steps in the Wnt signaling pathway (Figure S1-1) illustrate this difference. In Reactome, the first step is the binding reaction of Wnt, Fzd and Lrp5/6, to form a new molecular entity, the Wnt-Fzd-Lrp5/6 complex. This complex is an input in the next reaction, in which the Wnt-Fzd-Lrp5/6 complex binds phosphorylated Dvl, to form a distinct complex of Wnt-Fzd-Lrp5/6-Dvl-pp. In GO-CAM, the first step in Wnt signaling is the “receptor ligand activity” of Wnt, which then positively regulates the “receptor activity” of Fzd and “coreceptor activity” of Lrp5/6.

Figure S1-1

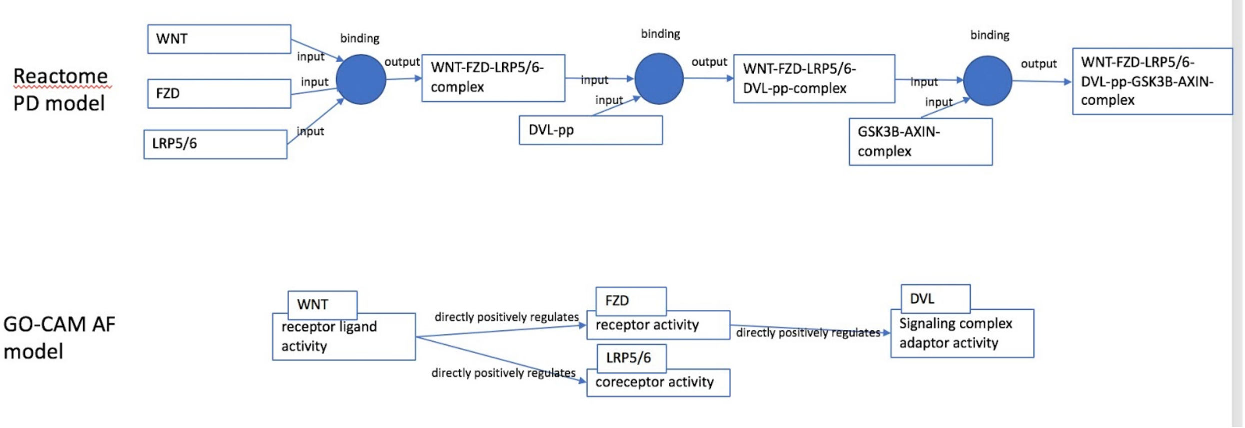

Successful conversion of Reactome reactions and relationships between them requires the establishment of rules to identify GO molecular functions that correspond to Reactome reactions, the entities that enable those molecular functions, and the causal relationships between Molecular Functions to assemble reactions into a pathway. A key advance in the work described here is the ability of the Pathways2GO process to capture causal relationships. Previous work mapping Reactome pathways to GO processes captured identities of participating physical entities and the functions they mediated, and used minimal numbers of these entities and functions to uniquely identify and distinguish pathways and corresponding GO processes (Hill et al. 2016). Causal relationships among the participants within Reactome pathways were lost in these simple mappings. GO-CAM captures these causal relationships. It does so, however, by representing them as an activity flow (AF) while they are annotated in Reactome as a process description (PD). These two complementary representations were developed by the Systems Biology Graphical Notation (SBGN) project (Le Nouvère et al. 2009). A PD representation of a pathway shows all the molecular processes and interactions taking place between biochemical entities,

and their outcomes. It depicts transitions of entities from one form to another as a result of different influences; thus, the temporal qualities of molecular events occurring in biochemical reactions are represented as in familiar drawings of metabolic pathways. As knowledge of a biological process increases, its PD representation increases rapidly in complexity. An AF representation avoids this complexity. Molecular details of processes are omitted and influences between activities are represented directly. Instead of displaying the details of biochemical reactions with process nodes and connecting arcs, AF diagrams show only influences such as ‘stimulation’ and ‘inhibition’ between the activities enabled by molecular entities. For example, a signal activity ‘stimulates’ a receptor activity, and the receptor activity in turn ‘stimulates’ the activity of an intracellular signaling adaptor protein.

To describe the action of a protein in an AF model of a reaction, it is necessary to identify an input entity that undergoes a transformation enabled by the activity of the protein. Successive transformations can be assembled into an AF model when the transformation in one reaction enables the activity required for a subsequent reaction. Enabling takes the form of generating a required input for that reaction. Reactions involving transformations of entities to novel chemical forms mediated by enzyme catalysts or to novel subcellular locations mediated by transport proteins readily fit this AF model: the Reactome PD controller maps to the GO-CAM AF active entity, and the PD reaction maps to the AF activity (Figure S1-2). This mapping is straightforward because small molecules, when covalently modified, are considered to be different molecules in both PD and AF: they are transformed into different entities. The causal relationship between sequential steps in a metabolic pathway is thus that the product of one reaction is the input of the other, or the MF of the first reaction ‘directly provides input for’ the MF of the second reaction.

Figure S1-2

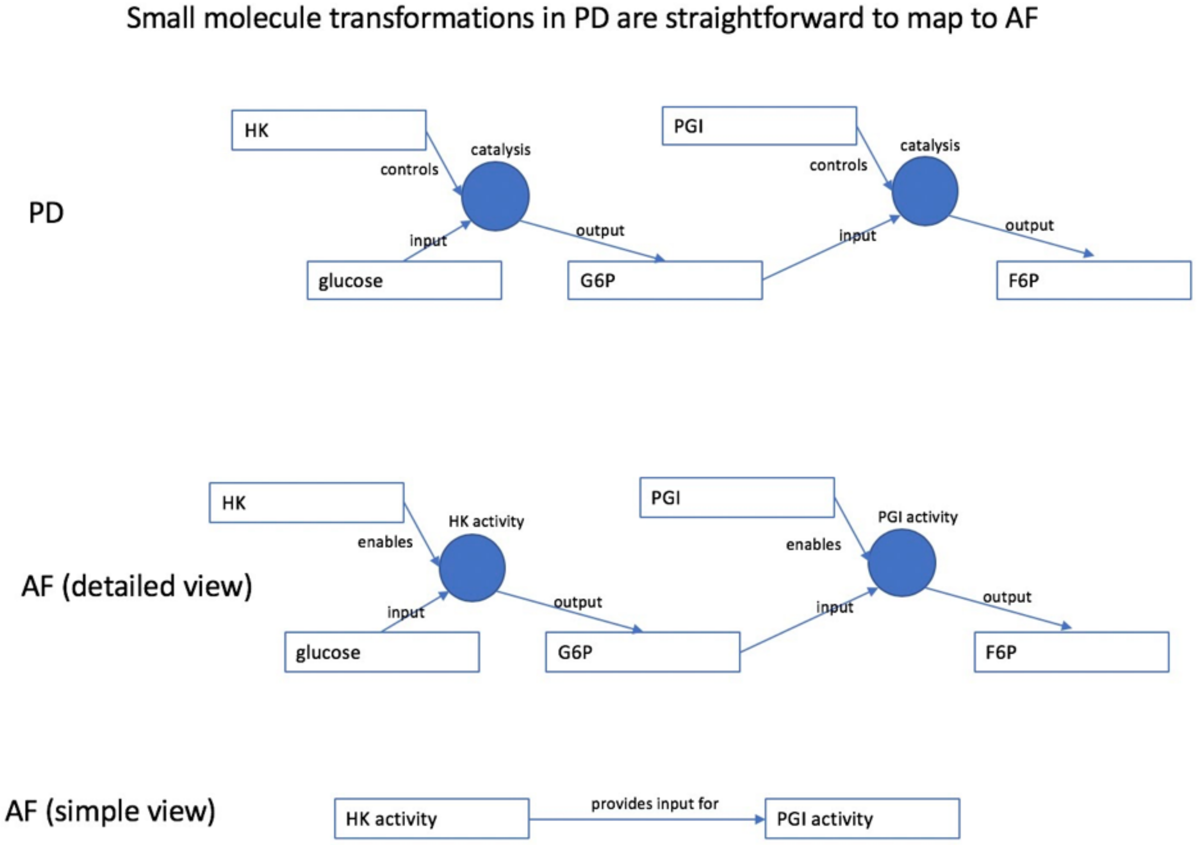

Reactions that influence the activity of a gene product, e.g. by activating a regulator or catalyst, are not straightforward to map, because of a fundamental difference in the PD and AF representations. In PD, a gene product entity is transformed into a different entity in the same way as a small molecule. This may be covalent (a protein is distinct from its phosphorylated form), spatial (a cytosolic protein is distinct from its nuclear form), or noncovalent (a protein complex is distinct from its constituent subunits). In AF, on the other hand, the focus is not transformations of a protein entity but rather its activity, and its modulation by an upstream activity. An upstream activity may also be covalent or noncovalent: a protein's activity can influence the activity of a second protein by covalently modifying it, transporting it, or binding it.

For a sequence of reactions that each have a controller (enzymatic or transport), the mapping between PD and AF is still fully specified. In this case, in PD the output of an upstream kinase reaction (e.g. a phosphoprotein) is the controller of a downstream reaction, which can be transformed into AF by asserting that the kinase activity of the upstream protein positively influences the activity of its downstream target (Figure S1-3).

Figure S1-3

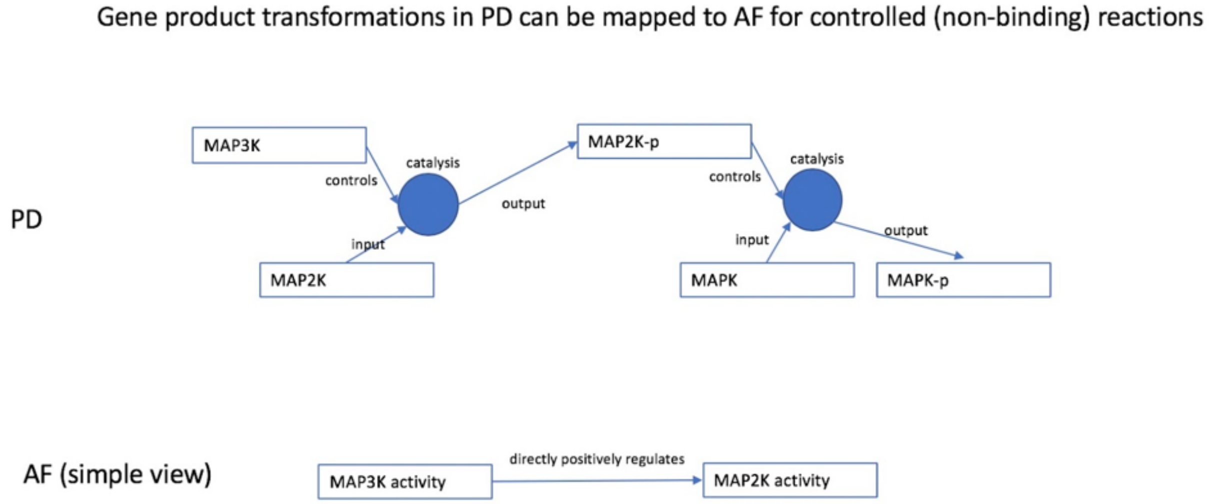

There is no such general PD-AF mapping when the upstream reaction is a binding reaction, because it is not possible to identify which of the inputs to the binding activity is the causal agent with respect to influencing downstream activities. That is, an AF representation requires that one input somehow influences the activity of the other to determine the directionality of causal influence from a binding reaction. This direction-of-influence information is not present in the standard PD representation.

We have instead made hybrid models of such binding reactions, adopting the PD model for binding reactions but the AF model for controlled reactions (Figure S1-4). A hybrid model is not strictly AF and thus loses the advantages of this representation, and violates the specific GO standards for representation of gene product function: an activity is to be associated with a specific gene product unless the activity is an emergent function that requires the products of multiple genes. For example, GO (and GO-CAM) treats the protein product of INSR as having "receptor activity," while in the hybrid model this activity is associated with a macromolecular complex comprised of the INSR and INS gene products, and the activity is incorrectly modeled as *enabled by* a complex rather than a gene product.

Figure S1-4.

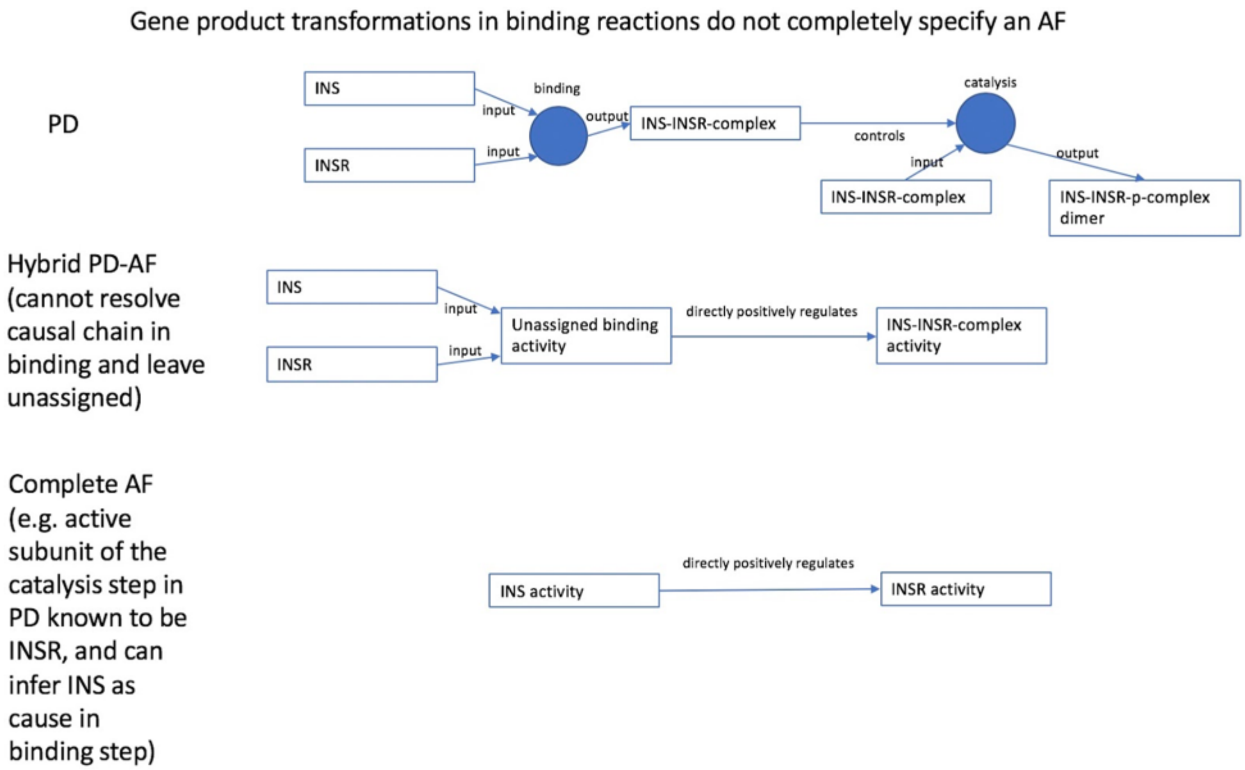

We therefore attempted to find additional information to allow us to infer causal directionality and define a fully causal model. An example of additional information provided by Reactome is the "active subunit" of a macromolecular complex. Currently, the active subunit is only specified for a subset of the reactions that are controlled by a macromolecular complex, and not for any binding reactions. If active subunit information were available, it would allow us to infer that one of the non-active input subunits preceding binding reaction to create the complex must be the upstream causal influence on the active subunit. In most cases, it is clear that Reactome curators had such causal activity flows in mind when annotating these reactions, demonstrated by the reactions' free text names.

Figure S1-5.

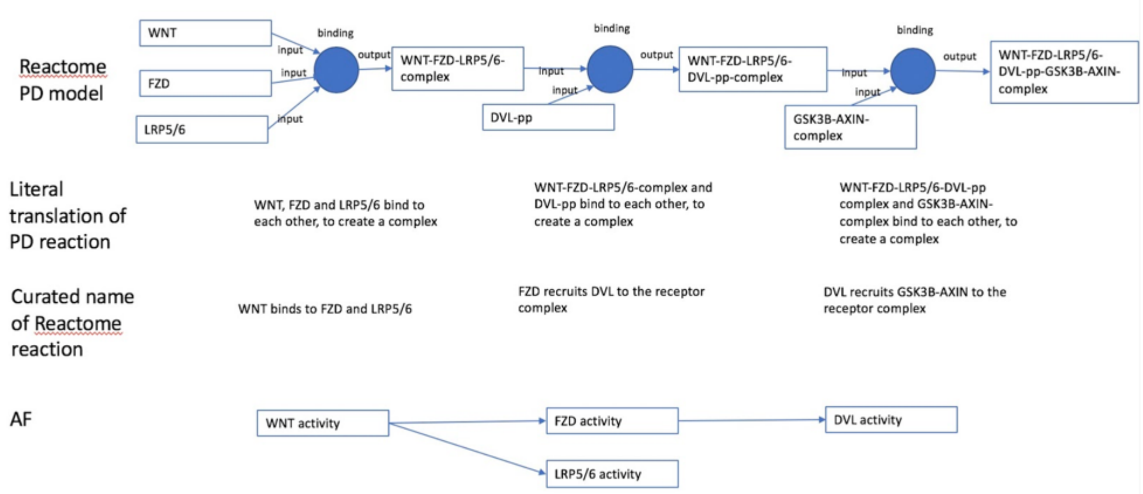

Figure S1-5 shows the first several steps of the WNT signaling pathway, and compares the literal interpretation of each Reactome reaction with the directionality implied by each reaction name. The reaction name clearly implies a causal directionality, e.g. “WNT binds FZD and LRP5/6” specifies WNT as the causal actor, but the PD model does not, stating simply that three proteins come together to form a complex. Reactome-derived GO-CAM models could in principle be edited manually to add this information post-import, but a better fix will be to annotate active unit information for Reactome binding reactions, to allow a fully specified GO-CAM model to be automatically constructed from the Reactome model.

References

Hill,D.P. et al. (2016) Modeling biochemical pathways in the gene ontology. Database (Oxford), baw126. doi: 10.1093/database/baw126.

Le Novère,N. et al. (2009) The Systems Biology Graphical Notation. Nat Biotechnol., 27, 735-741.

Thomas,P.D. et al. (2019) Gene Ontology Causal Activity Modeling (GO-CAM) moves beyond GO annotations to structured descriptions of biological functions and systems. Nat Genet., 51, 1429-1433.

Vastrik,I. et al. (2007) Reactome: a knowledge base of biologic pathways and processes. Genome Biol., 8, R3.

Supplement 2 - detailed Pathways to GO conversion procedure

The current procedure to generate a GO-CAM from a Reactome pathway expressed in the BioPAX (Level 3) exchange format is described here. The conversion software is freely available at https://github.com/geneontology/pathways2GO

Generate or identify an OWL ontology that contains a class representation of each of the physical entities in the BioPAX file.

The ontology for the Reactome conversion is called REACTO and is available at http://purl.obolibrary.org/obo/go/extensions/reacto.owl. For each entity:

a) **Use the BioPAX type assigned to the entity** {Physical Entity, Protein, Complex, DNA, RNA, SmallMolecule} and any available Xrefs (cross references) to assign the most specific applicable term from the GO-CAM ontology collection as **a superclass** of the new class.

- For proteins, the superclass is a term from the NEO ontology (https://github.com/geneontology/neo) that also corresponds to a UniProt entry such as https://www.uniprot.org/uniprot/P05062 for human fructose-bisphosphate aldolase B.
- These classes are subclasses of CHEBI:Protein http://purl.obolibrary.org/obo/CHEBI_36080
- For complexes, the superclass is always GO:Protein-Containing Complex (GO:0032991) as Reactome does not provide Xrefs to shared complex ontology terms.
- For DNA, the superclass is CHEBI:DNA http://purl.obolibrary.org/obo/CHEBI_4705
- For RNA, the superclass is CHEBI:RNA https://www.ebi.ac.uk/chebi/searchId.do?chebiId=CHEBI:33697
- For small molecules, the superclass is the CHEBI root term ‘chemical entity’ http://purl.obolibrary.org/obo/CHEBI_24431

b) **Capture the location** of the entity using the BioPAX:cellularLocation annotation. Locations in Reactome-provided BioPAX pathways are instances of classes in the GO cellular component (CC) ontology. This is captured in OWL with an axiom indicating that the class is a subclass of entities located in the GO CC term. E.g. (subclass of ('located in' some GO_0005829 (cytosol))).

c) **Capture the taxon** for the gene products and complexes using a subclass axiom, e.g. for human gene products (subclass of ('only in taxon' some OBO:NCBITaxon_9606)).

d) **Capture the canonical record** for each entity. The canonical record is an ontology term from the standard ontology collection used for GO-CAMs. It is either equivalent to or, more typically, a superclass of the entity term. This term is used when GO-CAMs that use this entity ontology are exported in forms such as GPAD that do not allow the use of imported ontologies to define their terms. This term will correspond to an entity at the deepest possible level of semantic

granularity. These records are added as annotation property assertions on the class.

- Canonical records for proteins are UniProt ids, e.g. http://identifiers.org/uniprot/P31947 - 14-3-3 protein sigma

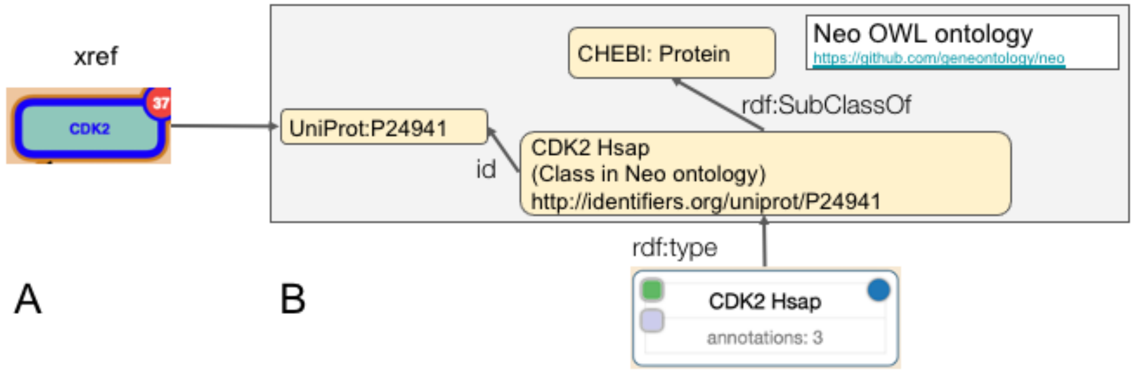

Figure S2-1. Canonical forms of proteins in Reactome (A) are mapped to GO-CAM REACTO instances (B) by means of their UniProt cross-references.

- Canonical records for DNA and RNA are Ensembl identifiers harvested from Xrefs, e.g. ENSEMBL:X03205
- Canonical records for small molecules are CHEBI terms, e.g. alpha-D-glucose 6-phosphate (https://www.ebi.ac.uk/chebi/searchId.do?chebiId=CHEBI:17665)
- Canonical records for complexes are all simply GO:'protein-containing complex' (https://www.ebi.ac.uk/QuickGO/term/GO:0032991) as mappings are not currently available to link Reactome complexes to more specific concepts in OBO ontologies.

e) **Capture the composition of protein complexes** using 'has component' axioms with cardinality constraints that capture stoichiometry, e.g. 'has component' exactly 1 'YWHAB dimer'. Here, 'YWHAB dimer' is another class in the entity ontology, itself a complex with composition (''has component' exactly 2 YWHAB'). Thus the ontology mirrors the recursive structure of complex definitions employed by Reactome.

f) **Capture protein modifications and locations** using 'has part' axioms and the PSI-MOD ontology (Montecchi-Palazzi et al. 2008), e.g., (subclass of ('has part' some omega-N-methyl-L-arginine) and (has_start value 12)).

g) **Represent sets using OWL union axioms**, e.g. ("glucokinase and hexokinases" is equivalent to (GCK or HK1 or HK2 or HK3)).

- For classes representing sets, add a canonical record annotation for each member of the set. E.g., for ("glucokinase and hexokinases", the canonical records are the UniProt ids for each of the proteins in the set, GCK, HK1, HK2, and HK3 (https://reactome.org/content/detail/R-HSA-450097) (Figure S2-3). When the canonical records are used for sets, for example to produce a GPAD export of a GO-CAM that uses the set, each canonical record is used in the same way it would be if there were only one entry. For example, if a model contained an assertion that the set

named ("glucokinase and hexokinases" enabled a molecular function MF1, then an export would contain the statements: GCK enables MF1, HK1 enables MF1, HK2 enables MF1, and HK3 enables MF1. In essence, the set construct is used by Reactome as a shorthand and this is preserved in the GO-CAM conversion process but for simpler formats it must be expanded.

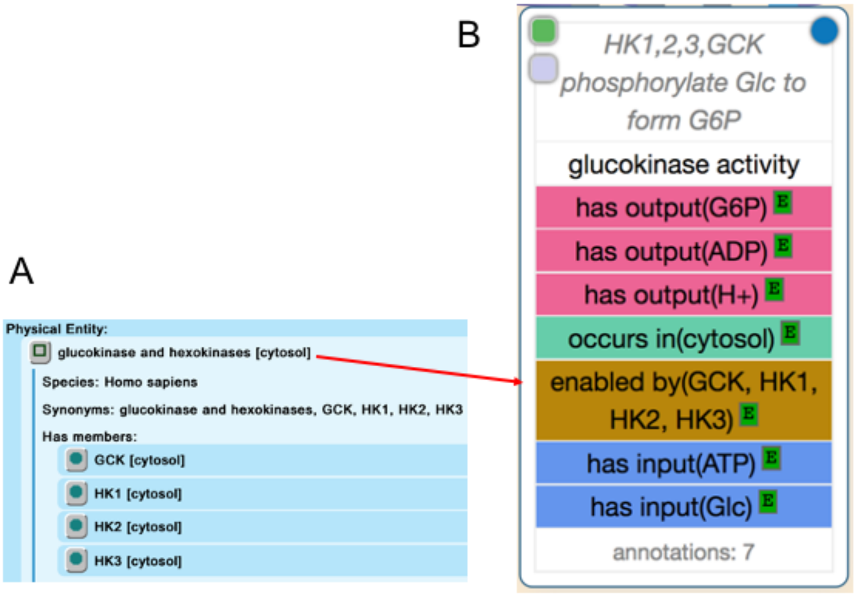

Figure S2-3. Physical entities are grouped into sets in Reactome (A) by the 'has members' relationship. The members of a set are related by a logical OR: when the set object is said to have a function, any member of the set is equally able to enact that function. These set properties are preserved in their logical definition in the REACTO ontology (B).

2) **Construct the OWL:individuals for the model with default types**:

   a) Create a biological process node with a GO:Biological Process type for each BioPAX:Pathway

   b) Create a molecular activity node with a Molecular Event (parent of Molecular Function) type for each BioPAX:BiochemicalReaction.

3) **Use provided BioPAX:RelationshipXrefs to add deeper classifications**:

   a) **BioPAX:Pathway** nodes are directly annotated with GO Biological Process terms. For example, the pathway node in the Reactome pathway "Signaling By BMP" provides a RelationshipXref link to the GO Class "BMP signaling pathway" (GO:0030509). The converter would thus assign the corresponding individual rdf:type GO:0030509.

   b) **BioPAX:BiochemicalReactions** that have controllers such as catalysts are linked to a BioPAX:Control node that may in turn have RelationshipXref annotations linking it to a GO Molecular Function term. Where these are available, they are used to provide a deeper type for the associated GO:Molecular Function individual in the GO-CAM. For example, the reaction alpha-D-glucose 6-phosphate ⇔ D-fructose 6-phosphate" has an associated BioPAX:Catalysis event that is annotated with a RelationshipXref to GO:0004347

(glucose-6-phosphate isomerase activity). Hence the GO-CAM would have a function node for this reaction with type GO:0004347 (Figure S2-4).

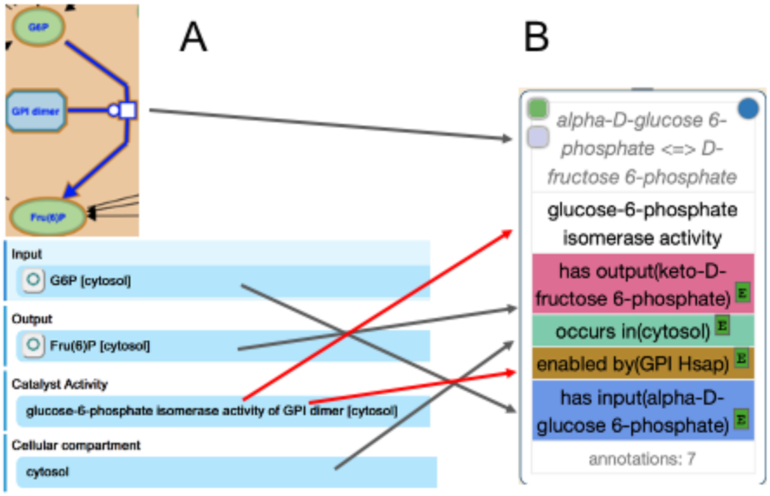

Figure S2-4. When Reactome associates a catalytic or transporter activity with a reaction and assigns a GO term (A), that term defines the type of the GO-CAM function node (B) with no information loss, illustrated here for the catalyzed conversion of alpha-D-glucose 6-phosphate to D-fructose 6-phosphate (B) (red arrows). Black arrows show mappings of other reaction attribute.

4) **Attempt to infer classes for individuals with no provided Xrefs**.

   a) Infer Molecular Function type for activity nodes

      ■ If a reaction has no curated Molecular Function

         ○ and is assigned an enabling entity

            ● Then assign it the type Molecular Function

   b) **Infer Transport activities (Figure S2-5)**.

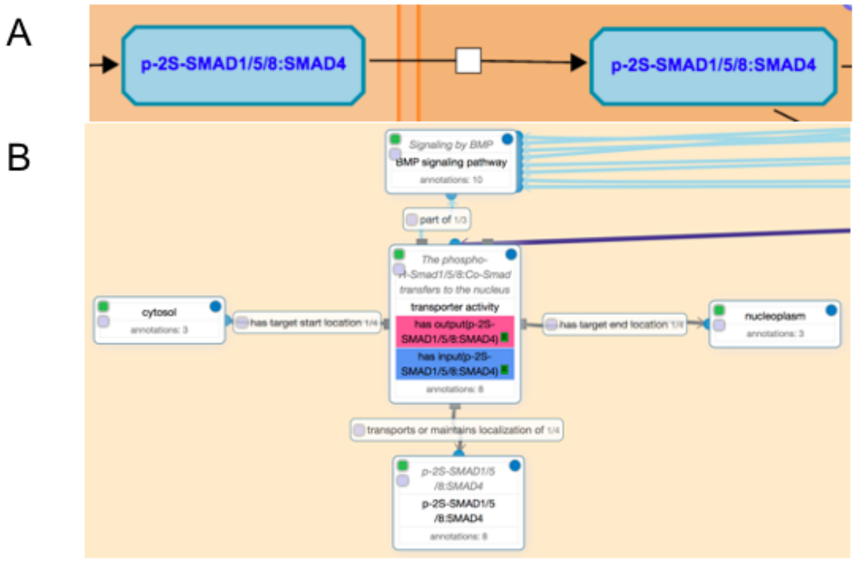

Figure S2-5. Inferring transport where no transport activity GO term is associated with a reaction. If such a reaction has the same physical entities as both inputs and outputs with the input and output entities in different locations, the reaction is inferred to be of the transport type (GO:0005215)

- If a reaction has no curated GO Molecular Function
    - and the input entities are the same as the output entities
    - and the input entities have different locations from the output entities
  - Then add type 'transporter activity' GO:0005215 (protein transporter activity GO:0140318 if input and output entities are protein.

5) **Link physical entities relevant to the representation of the GO-CAM molecular function to the model**.
   a) When BioPAX:BiochemicalReactions have catalysts, the physical entity in the 'controller' slot in the BioPAX:Control node is typed with a physical entity term from the physical entity ontology created in step 1 and linked to the corresponding molecular function node in the GO-CAM using the RO:'enabled by' (RO:0002333) property. For Reactome models, catalysts can either be proteins or complexes.
      - When the catalyst is a complex, Reactome sometimes provides an indication of which protein is the active unit within the complex. When this information is present, the active protein is added to the GO-CAM as the enabler of the molecular function. When there is no active unit annotation, a node corresponding to the complex is created and linked to the molecular function node with the 'enabled by' relation.
        - The active-unit information cannot be represented in the BioPAX model. For this project, Reactome added references to the active protein within comments on the associated BioPAX:Control nodes. E.g. rdfs:comment activeUnit: #Protein436 links a Control node with a protein complex as its Controller to the specific protein within that complex that is the active unit.
   b) All reactions have substrates and products, indicated in BioPAX using the properties 'left' and 'right' along with a conversionDirection annotation e.g. 'LEFT-TO-RIGHT'. These physical entities are added to the GO-CAM using the "has input" (RO:0002233) and "has output" (RO:0002234) properties. They may be physical entities of any kind: complexes, proteins, small molecules, RNA, or DNA.

6) **Infer location information assertions for all activity nodes**
   a) The locations of the physical entities in a reaction are used to establish where the reaction occurs.
      - If all the entities are in the same location (Figure S2-4),
        - Then the function node is linked to that location using the "occurs in" (BFO:0000066) relation from the Basic Formal Ontology (a super property imported by the Relation Ontology). Reactome makes use of the cellular component branch of the GO to represent location.
      - If a reaction has an enabler, the reaction location is assigned to be that of the enabler, regardless of the locations of participating physical entities.

These additional locations are preserved as attributes of the physical entities
If the input entities are the same as the output entities, the input entities have different locations from the output entities, and the type of the molecular function term associated with the activity is a subclass of ‘transporter activity’

- Then the function node is linked to the locations via the ‘has target end location’ (RO:0002339) and ‘has target start location’ (RO:0002338) relations.
- In addition, the physical entity that is transported is linked to the activity with the ‘transports or maintains localization of’ (RO:0002313) relation.

7) **Infer causal relations between molecular function nodes.** BioPAX captures connections between reactions using BioPAX:PathwaySteps. In contrast to the GO-CAM activity flow model, which identifies different types of relationships between activities, BioPAX only captures the order of events. Information about how one reaction affects another must be inferred based on the inputs and outputs of the connected reactions. The first step in the conversion process is to translate all of the ‘nextStep’ relations between reactions into RO ‘causally upstream of’ (RO:0002411) assertions linking activity nodes in the GO-CAM. Note that this relationship does not imply positive or negative influence, only that the activities are related to one another in a causal chain. Once this basic network of activities is established, several rules are applied to further specify the nature of the causal relationships between activities. Note that these rules are applied to the GO-CAM model itself, after it has been generated from the BioPAX and, as such, could be applied to any GO-CAM model. More than 30% of the causal relationships between reactions in Reactome occur between reactions in different pathways. The conversion framework produces one GO-CAM for each pathway and currently has no ability to handle links from one node of a GO-CAM to another node in a different GO-CAM. In order to address this limitation, when a causal relationship exists that crosses a pathway boundary, a copy of the upstream converted reaction is added to the pathway containing the downstream reaction. This introduces a redundancy as the system as a whole now contains two activity units (one per pathway) for the same Reactome reaction. However, this redundancy is reduced through the use of the same internal identifiers for the reaction in both converted pathways. Through this unique identifier approach, when the whole knowledgebase is queried via a SPARQL endpoint, the reaction nodes in the different pathways can be merged. This allows the full causal graph to be captured and queried over in a unified way. It also provides us with an automated flag for examination of reactions that should be added to more than one pathway. Future work in the GO-CAM software framework should better enable this kind of activity node linking and re-use.

   a) **Infer RO ‘directly provides input for’ (RO:0002413)**
      - If Reaction 1 has an output that is an input to Reaction 2,
      - And Reaction 1 is causally upstream of Reaction 2
         - Then Reaction 1 directly provides input for Reaction 2

   b) **Infer RO ‘directly positively regulates’ (RO:0002629) (Figure S2-6)**
      - If Reaction 1 has an output that enables Reaction 2

- And Reaction 1 is causally upstream of Reaction 2
  - Then Reaction 1 directly positively regulates Reaction 2

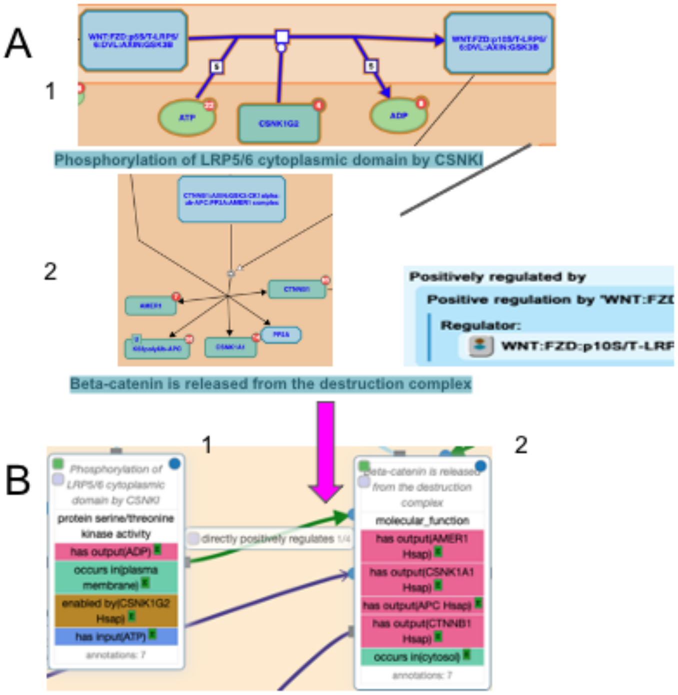

Figure S2-6. Inferring regulation. If an output of a Reactome reaction positively or negatively regulates a second reaction (A) - here, the output of reaction 1 positively regulates reaction 2 - a directly_positively (or negatively) regulates relationship is created between the two corresponding GO-CAM activity units

c) **Infer directly provides input for - new binding activity node - directly regulates** (Figure S2-7)

- If Reaction 1 has an output that is a controller (that is not a catalyst) of Reaction 2
- And Reaction 1 is causally upstream of Reaction 2
  - Then create new activity node B1 with type Binding
  - Assert that B1 is part of the main Biological Process node representing the pathway
  - Add the controlling entity as an input to the new Binding node
  - Add Reaction 1 directly provides input for B1
  - Add B1 directly (positively or negatively) regulates Reaction 2

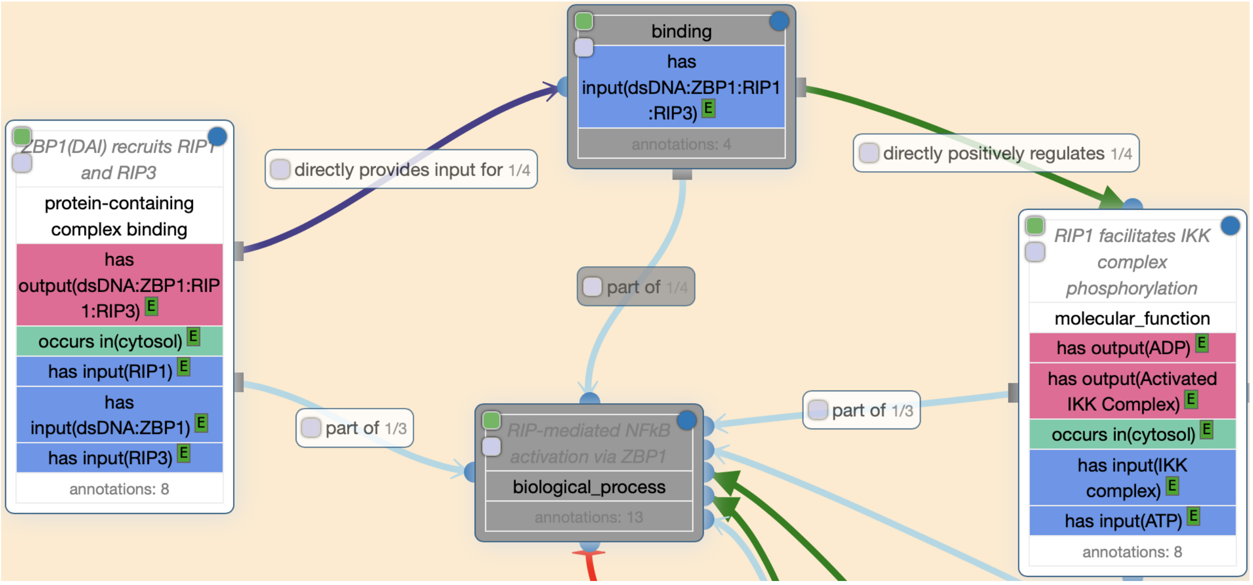

Figure S2-7. Inference of binding activity.

8) **Convert non-catalytic entity regulators to binding activity nodes**. In contrast to the GO-CAM model, which strictly focuses on the activities of gene products, Reactome models the regulatory effects of other kinds of molecules on reactions. For example, the Glycolysis pathway in Reactome asserts that ADP positively regulates the reaction D-fructose 6-phosphate + ATP → D-fructose 1,6-bisphosphate + ADP. To put this in the GO-CAM framework, which does not model direct regulation by physical entities, it is necessary to generate an activity node to capture the information and link that node to the reaction being regulated. The rules for representing regulation by a physical entity other than a gene product follow and the results are presented in Figure S2-2.

- If a reaction is positively or negatively regulated by a physical entity and that entity is not a catalyst, then:
  - Create a new binding node
  - Add the regulator as an input
  - Add a positive or negative regulatory relationship from the new binding node to the reaction node as indicated in the BioPAX Controller element.
  - Make the Binding node part of a root Biological Process node.
  - Make the Biological Process node regulate the pathway node that the reaction is a part of.
  - If the original reaction is enabled by something,
  - Then add the enabler as an input to the new Binding node.

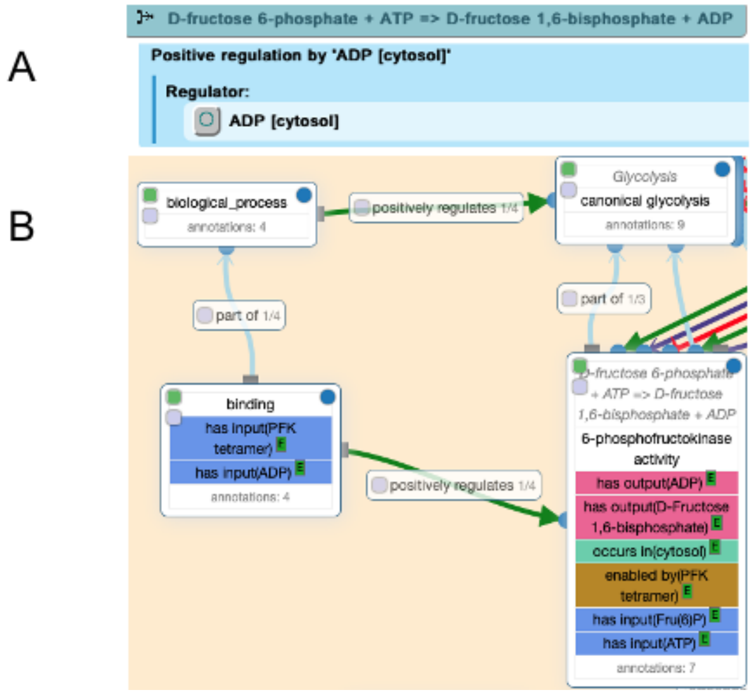

Figure S2-8. Representing positive regulation of PFK enzyme activity by the small molecule ADP in Reactome (A) and in a GO-CAM model (B).

Filters

As the GO focuses on 'normal' biology, all pathways under the 'Disease' branch of the Reactome pathway hierarchy were eliminated from the analysis. This reduced the total number of pathways from 2362 to 1815. In addition, some pathways representing normal biology contain reactions that involve drugs (e.g. CES1 trimer hydrolyses ACEI pro-drugs to ACEIs in the pathway Metabolism of Angiotensinogen to Angiotensins). In these cases, the pathway is preserved in the translation but the reactions involving drugs are taken out. Note that the GO-CAM framework is capable of representing both disease and drug biology. The decision to keep this information out of the conversion process reflects the goal of using the conversion in the automated import of Reactome content into the GO knowledge base, respecting the current scope of GO.

Implementation details

The conversion was implemented as a Java program that leverages:

1. the PaxTools library (Demir et al. 2013) for parsing the BioPAX level 3 files provided by Reactome
2. the OWL API [https://github.com/owlcs/owlapi] for constructing and exporting the GO-CAM models.
3. The Jena RDF library [https://jena.apache.org] for implementation of certain conversion rules and tests.
4. The Arachne reasoner (https://github.com/balhoff/arachne) for testing logical consistency of the models (Balhoff et al. 2018)

5. The shex-java library [https://github.com/iovka/shex-java] for testing that the GO-CAM models adhere to the GO-CAM specification [https://github.com/geneontology/go-shapes].

The GO-CAM models produced by the program are serialized in the Terse RDF Triple Language (.ttl) format (https://www.w3.org/TR/turtle/). This format is one of several equivalent serializations of RDF. Others include RDF/XML, N-triples, and N3. Turtle was selected as it is relatively easy to read and is well supported by semantic web libraries and tools such as Protege.

Although the models can be processed by any RDF-capable software, they are best used and understood as small OWL ontologies. They can be examined in Protege, reasoned over with tools such as Arachne, and queried using either OWL-DL or SPARQL tools. Additional information about uses of the knowledge in the GO-CAM models can be found at http://geneontology.org/docs/gocam-overview/.

In addition to the ontologies described in the main text such as the Gene Ontology, GO-CAMs also make use of a small application ontology called 'lego' that is used to add useful metadata such as the x and y positions of nodes within the noctua editor. This ontology is not used anywhere else.

Reference

Balhoff,J.P. et al. (2018) Arachne: an OWL RL reasoner applied to Gene Ontology Causal Activity Models (and beyond). Presented at the 17th International Semantic Web Conference (ISWC 2018). Monterey, CA, USA: Zenodo. http://doi.org/10.5281/zenodo.2397192.

Demir,E. et al. (2013) Using biological pathway data with paxtools. PLoS Comput Biol., 9, e1003194.

Montecchi-Palazzi,L. et al. (2008) The PSI-MOD community standard for representation of protein modification data. Nat Biotechnol., 26, 864-866.
